# Restoring global offset and lower limb length with a 3 offset option double-tapered stem

**DOI:** 10.1186/s12891-020-03674-8

**Published:** 2020-10-02

**Authors:** Stefano Biggi, Lorenzo Banci, Riccardo Tedino, Andrea Capuzzo, Gabriele Cattaneo, Stefano Tornago, Andrea Camera

**Affiliations:** 1 Clinica Città di Alessandria – Policlinico di Monza, via Moccagatta 30, 15122 Alessandria, AL Italy; 2 Fondazione Lorenzo Spotorno – Onlus, Albenga, SV Italy; 3 Permedica S.p.A, Merate, LC Italy

**Keywords:** Total hip Arthroplasty, Offset, Global offset, Leg length, Hip parameters

## Abstract

**Background:**

A proper restoration of hip biomechanics is fundamental to achieve satisfactory outcomes after total hip arthroplasty (THA). A global hip offset (GO) postoperatively reduction of more than 5 mm was known to impair hip functionality after THA. This study aimed to verify the restoration of the GO radiographic parameter after primary THA by the use of a cementless femoral stem available in three different offset options without length changing.

**Methods:**

From a consecutive series of 201 patients (201 hips) underwent primary cementless THA in our center with a minimum 3-year follow up, 80 patients (80 hips) were available for complete radiographic evaluation for GO and limb length (LL) and clinical evaluation with Harris hip score (HHS). All patients received the same femoral stem with three different offset options (option A with – 5 mm offset, option B and option C with + 5 mm offset, constant for each sizes) without changing stem length.

**Results:**

Mean GO significantly increased by + 3 mm (*P* < 0.05) and mean LL significantly decreased by + 5 mm (P < 0.05) after surgery, meaning that postoperatively the limb length of the operated side increased by + 5 mm. HHS significantly improved from 56.3 points preoperatively to 95.8 postoperatively (*P* < 0.001). Offset option A was used in 1 hip (1%), B in 59 hips (74%) and C in 20 hips (25%).

**Conclusions:**

The femur is lateralized with a mean of + 5 mm after surgery than, the native anatomy, whatever type of stem was used. Thus, the use of this 3-offset options femoral stem is effective in restoring the native biomechanical hip parameters as GO, even if 2 offset options were considered sufficient to restore GO.

## Background

A proper restoration of joint biomechanical parameters is fundamental to achieve satisfactory outcomes in terms of hip functionality and patient’s quality of life after total hip arthroplasty (THA).

The hip abductor muscles lever arm, which is mechanically the distance between the center of rotation of the hip and the insertion point of the muscles, is in fact strictly dependent by implant positioning which determines the reconstruction of the center of rotation, as well as the global hip offset or global femoral offset (GO) [1]. GO is the distance from the anatomical axis of the femur to the medial margin of the acetabulum and it is usually defined as the sum of the femoral offset (FO) and the acetabular offset (AO) [1]. FO is defined as the distance from the center of rotation of the femoral head to the anatomical axis of the femur [2]. AO is defined as the distance from the center of rotation of the femoral head to the perpendicular line passing through the medial edge of the ipsilateral teardrop [3].

When studying hip parameters restoration after primary THA, GO is more reliable as joint parameter than considering only FO because it takes into account also the acetabular cup placement.

It has been showed how a GO reduction of more than 5 mm is associated with less abductor muscle strength and decreased functional results after THA [4]. Femoral stems are usually available with different offset versions but not always this can be a suitable solution as these different options often change, not only the stem offset but also, at the same time, the stem length and, thus, limb length (LL). A traditional cementless double-tapered straight femoral stem available with three different offset options has been shown to be able to restore hip offset even if LL discrepancy is not always minimized postoperatively [5, 6].

This study aimed to verify the restoration of the ipsilateral GO and LL radiographic parameter after primary THA by the use of a cementless femoral stem available with three different offset options which do not change the stem length. Thus, the study hypothesis, that we want to reject, is that postoperative GO decreased by more than 5 mm in comparison to preoperative GO.

## Methods

### Study cohort

All consecutive patients who underwent primary THA using the same femoral stem from 2014 to 2015 at authors’ previous institution (the Department of Prosthetic Surgery of the Santa Corona Hospital of Pietra Ligure – Italy) were identified through the hospital database.

If deemed suitable according to the inclusion / exclusion criteria of the study protocol, patients were contacted by telephone by one of the authors and invited to participate in the study by attending a follow-up visit.

Inclusion criteria were defined as diagnosis of unilateral primary or secondary osteoarthritis, avascular osteonecrosis of femoral head, mild dysplasia of the hip (Crowe I-III), cementless primary THA with the same femoral stem at the same center, same senior surgeon, 3-year minimum follow-up. Exclusion criteria were defined as history of hip surgery before THA, diagnosis of severe dysplasia of the hip (Crowe ≥IV) or femoral neck fracture, revision THA, cemented femoral stem, patients not able to walk, missing preoperative or postoperative radiographs.

Overall, 201 patients (201 hips), who met all inclusion/exclusion criteria, were identified to be suitable to be enrolled in this study. Out of these 201 patients, 7 patients were dead for causes unrelated to the surgery and 40 patients were lost to follow-up, being unable to contact them by phone or mail. Thus, 154 patients were contacted to attend a follow-up visit: 80 patients were visited at the last follow-up with available preoperative and postoperative radiographs and the remaining 74 patients were called only by phone but without follow-up visit and postoperative radiographs.

At the follow-up visit, an objective evaluation of the operated hip was carried out with the Harris hip score (HHS) [7] completed on patient’s current state of health, as well as with a radiographic evaluation and measurements for specific radiographic parameters. Any intraoperative and postoperative complication related to the implant and implant failure with components revision were recorded for femoral stem and implant survivals. All patients signed an informed consent form for their personal data recording and use. The study protocol has been approved by the competent ethics committee (protocol number AslAL.Orto.19.01).

### Prosthetic implant

Before surgery, preoperative hip templating was always performed. All surgical procedures were performed by the senior author (AC), using a posterolateral approach, without external rotators reconstruction and the patient placed in lateral position [8].

**Fig. 1:**
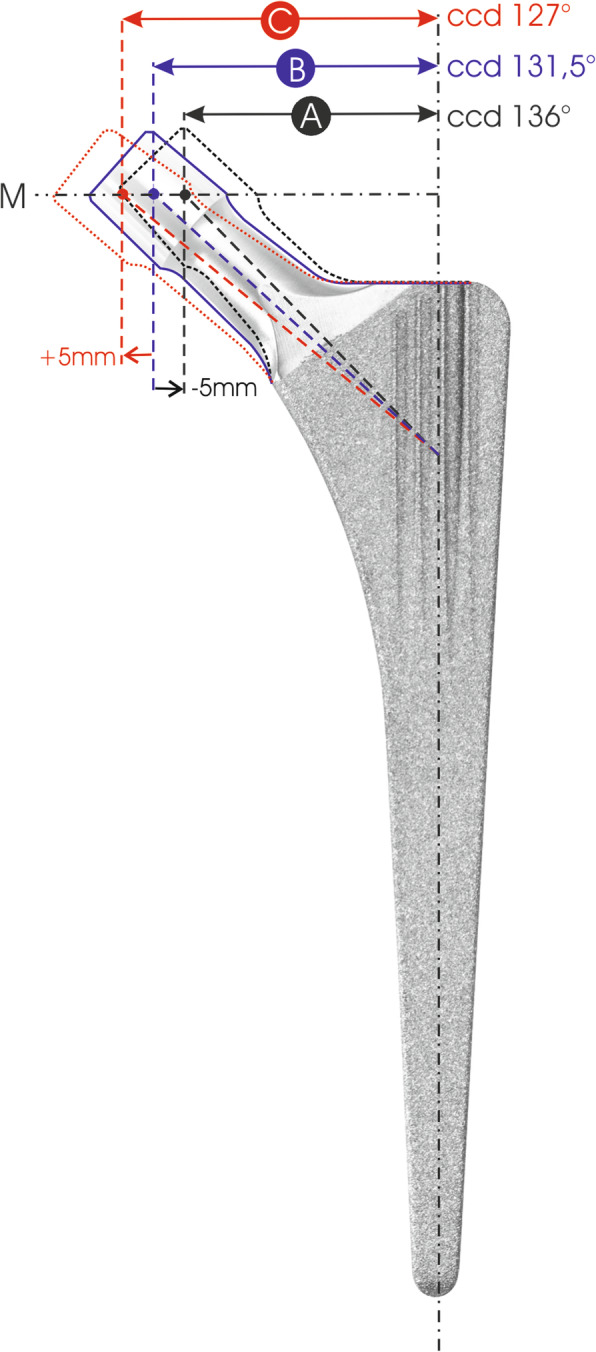
The Synthesis femoral stem (Permedica S.p.A., Merate, Italy) investigated in the study. The Synthesis stem is a cementless, sandblasted, double-tapered, straight, CLS Spotorno-like design. The Synthesis stem has three different offset options (offset A, B and C) which do not change the stem length. Offset B stem has 0 mm offset and 131° CCD angle. Offset A stem has −5 mm offset and 136° CCD angle. Offset C stem has + 5 mm offset and 127° CCD angle. The offset is constant for each stem size

The same femoral component, the Synthesis femoral stem (Permedica S.p.A., Merate, Italy), was used in all cases. Synthesis is a conventional cementless sandblasted titanium femoral stem with a double-tapered straight wedge profile and a rectangular cross-section. Although sharing a similar design with the classic CLS Spotorno stem (Zimmer Inc. Warsaw, IN), the Synthesis stem has three different offset options for each of the 13 sizes (stem options A, B and C) without changing stem length, unlike the CLS Spotorno stem [9]. Compared to type B stem (standard option with 131° CCD angle and 0 mm offset lateralization), type A stem has a decreased offset by − 5 mm, constant for each size, and type C stem has an increased offset by + 5 mm, constant for each size (Fig. 1).

The assessment of the correct offset option was performed during preoperative templating and then confirmed intraoperatively with trial components, considering hip morphotype and the possibility of correction of both limb length and hip offset.

Zirconia-reinforced alumina matrix (Biolox® Delta – CeramTec GmbH) 32 mm-diameter femoral head coupled with a highly cross-linked ultra-high molecular weight polyethylene liner and a cementless porous tantalum acetabular shell (Zimmer Biomet) was used in most of the hips.

### Radiographic analysis

Standard anteroposterior (AP) radiographs were taken preoperatively and postoperatively at the latest follow-up with the patient in supine position and both legs with 15° of internal rotation. The X-ray beam was centered on the symphysis pubis. All radiographic measurements were taken twice, at different time points, on digital AP radiographs of the pelvis by the same author (SB), who was not involved in index surgery. Recorded values were the average of the two measurements.

Femur morphology was defined according to Dorr classification [10] and by measuring the Flare index [11] on the preoperative AP pelvis radiograph.

Femoral offset (FO), defined as the perpendicular distance from the center of rotation of the femoral head to the anatomical femoral axis [12], was measured preoperatively and postoperatively on AP pelvis radiographs. Acetabular offset (AO), defined as the perpendicular distance from the center of rotation of the femoral head to the line passing through the medial edge of the ipsilateral teardrop perpendicular to the line passing through the lower margins of the ischial tuberosity [13], was measured preoperatively and postoperatively on the AP pelvis radiographs. GO was measured as the sum of FO and AO [14]. GO difference (ΔGO) was defined as postoperative GO – preoperative GO. Lower limb length (LL), defined as the distance between the medial apex of the ipsilateral lesser trochanter and the line passing through the lower margins of the ischial tuberosity [15], was measured preoperatively and postoperatively on AP pelvis radiographs. LL difference (ΔLL) was defined as postoperative LL – preoperative LL.

Moreover, GO and LL were measured also on the contralateral hip to assess GO and LL discrepancies between the operated side and the contralateral side after surgery.

**Fig. 2:**
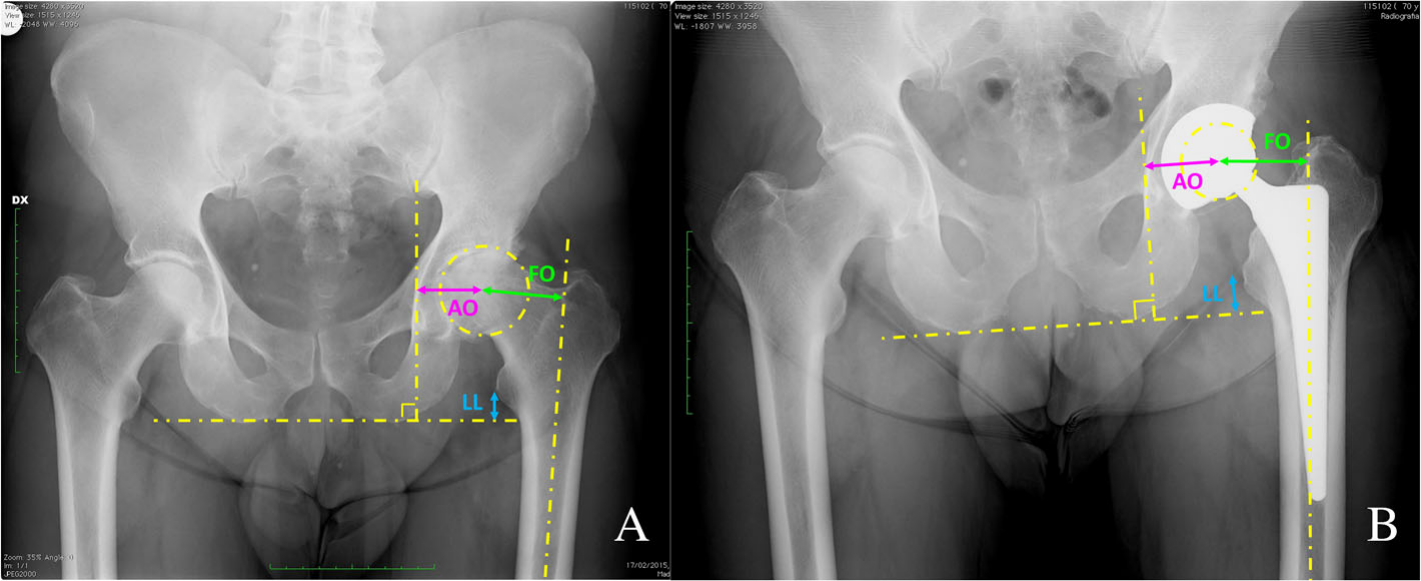
Measurements of the hip biomechanical radiographic parameters by using Horos Viewer software for MAC at preoperative (**a**) and follow-up time (**b**). The global hip offset (GO) was defined by the sum of the femoral offset (FO) and the acetabular offset (AO). Limb length (LL) was defined as the distance between the medial apex of the ipsilateral lesser trochanter and the line passing through the lower margins of the ischial tuberosity

GO and LL distances were measured by using Horos Viewer software for MAC (Fig. 2).

Stem positioning, stem distal migration (subsidence), periprosthetic femoral fractures, periprosthetic endosteal bone formation (spotwelds), cortical hypertrophy, osteolysis, stress-shielding, pedestal formation under the tip of the stem and component stability were assessed on radiographs [16].

Stem subsidence was defined as femoral stem distal migration greater than 2 mm seen on the last AP radiograph in comparison to the immediately postoperative AP radiograph [17].

In particular, the radiolucency lines and osteolysis areas were reported and evaluated according to the Gruen method [18] on the femoral side.

### Other assessments

Any complication occurred was recorded at the patient follow-up examination or at the telephone call.

The survival of the stem and the overall implant survival were analysed according to Kaplan Meier method [19], setting femoral component revision and any other components revision due to any reason and due to aseptic loosening as the end-point.

### Statistical analysis

Descriptive statistical analysis was carried out to summarize the study results for each parameter considered. Continuous variables were reported by mean, maximum and minimum values and standard deviation. Dichotomous variables were reported by number and percentage. Analysis of continuous variables were performed with the t-Student test, whereas analysis of dichotomous variables with the chi-square test. Statistical significance was set with a *p* < 0.05.

The study hypothesis to reject, was that ΔGO resulted < − 5 mm. Study alternative was that ΔGO resulted > 5 mm. Sample size analysis for 2 dependent groups (preoperatively vs postoperatively) using a difference of 5 mm for the primary endpoint variable (ΔGO) with a SD of 6 mm and alpha error of 0.05 required at least 15 subjects to include.

Statistical analysis was carried out with the MiniTab® 17.2.1 statistical software.

## Results

Mean (SD) age at surgery of the assessed patients was 72 (8) years. Mean (SD) BMI was 26.9 (3.9), 26 patients (32%) were male and 54 patients (68%) were female. Mean (SD) follow-up was 4.4 (0.6) years, ranging between 3.2 and 5.7 years. Diagnosis was primary osteoarthritis in 63 hips (82%), avascular osteonecrosis in 8 hips (10%), femoral neck fracture sequelae in 4 hips (5%), mild hip dysplasia in 2 hips (1%), others in 2 hips (1%). Femur morphology was classified as type A in 26 hips (32%), type B in 51 hips (64%), type C in 3 hips (4%), according to Dorr classification. Mean (SD) Flare index was 3.66 (0.70), ranging from 1.92 to 5.78.

The type of Synthesis femoral stem used was A type in 1 hip (1%), B type in 59 hips (74%), C type in 20 hips (25%).

Mean GO distance significantly increased by + 3 mm after surgery (*P* < 0.05). ΔGO was not < − 5 mm, but within ±5 mm range, then the alternative hypothesis was accepted and the null hypothesis was rejected (Table 1).

**Table 1:** Analysis of the hip parameters compared between preoperative radiographs and postoperative radiographs. n.s. not statistically significant. n.a., not applicable

Radiographic parameter	Preoperative Mean ± SD (range)	Postoperative Mean ± SD (range)	***p***-value
GO (all hips)	79.3 ± 14.7 (57–168) mm	81.8 ± 19.1 (58–230) mm	0.014
GO (offset B stem, 59 hips)	77.8 ± 15.8 (57–168) mm	80.5 ± 21.6 (58–230) mm	0.048
GO (offset C stem, 20 hips)	84.2 ± 10.1 (58–100) mm	86.3 ± 8.6 (74–110) mm	n.s.
GO (all contralateral hips)	80.8 ± 9.3 (60–115) mm	n.a.	
LL (all hips)	17.4 ± 10.7 (1–83) mm	11.9 ± 9.8 (1–60) mm	0.0001
LL (offset B stem, 59 hips)	18.1 ± 11.7 (1–83) mm	11.5 ± 8.6 (1–46) mm	0.0001
LL (offset C stem, 20 hips)	15.8 ± 7.0 (4–27) mm	13.9 ± 12.6 (1–60) mm	n.s.
LL (all contralateral hips)	12.0 ± 9.7 (1–60) mm	n.a.	

Mean LL distance significantly decreased by − 5 mm after surgery (*p* < 0.05), meaning that postoperatively the limb length of the operated side increased by + 5 mm (Table 1).

GO measured preoperatively in hips treated with type B stem was significantly lower (*p* < 0.043) in comparison with GO measured preoperatively in hips treated with type C stem (Table 2). It means that the choice of the stem type used (type B or type C) is actually dependent on preoperative hip offset. GO measured postoperatively did not differ (*p* > 0.05) between hips with type B and type C stem (Table 2). Thus, the femur was lateralized after surgery at least as, or more than, the native anatomy, whatever type of stem was used.

**Table 2:** Analysis of the radiographic parameters and the HHS compared between hips treated with offset B stem and hips treated with offset C stem

Parameter	Offset B stem (59 hips) Mean (SD)	Offset C stem (20 hips) Mean (SD)	*p*-value
GO preop	77.8 (16.0) mm	84.2 (10.1) mm	0.043
GO postop	80.5 (21.8) mm	86.3 (8.6) mm	n.s.
Δ GO	2.7 (10.0) mm	2.1 (4.7) mm	n.s.
LL preop	18.1 (11.7) mm	15.7 (7.0) mm	n.s.
LL postop	11.6 (8.6) mm	13.9 (12.6) mm	n.s.
Δ LL	−6.5 (11.1) mm	−1.8 (12.3) mm	n.s.
HHS postop	96.2 (6.1)	95.2 (8.1)	n.s.

LL measured postoperatively did not differ (*p* > 0.05) between hips with type B stem and those with type C (Table 2). LL was not affected by the type of stem (which actually does not modify LL) but rather depends on the size of the stem used and the positioning and size of the cup.

HHS significantly improved from 56.3 points preoperatively to 95.8 postoperatively (*p* < 0.001).

There was no significant difference in postoperative HHS between patients with type B stem and patients with type C stem (respectively, 96.2 vs 95.2, *p* > 0.05).

There were no significant differences for GO and LL between the operated side and the contralateral side after surgery, with a GO and a LL discrepancies of + 1 mm and − 0.1 mm, respectively (Table 1).

No stem subsidence was found at last follow-up. Presence of slight radiolucencies (< 2 mm) due to proximal bone remodeling was observed mostly in zone 1 in 50 hips (62%) and zone 7 in 11 hips (14%). No femoral cortical hypertrophy was seen in any radiograph. Pedestal formations were present in 2 hips. Spotwelds were noted in 19 (24%) hips predominantly in zone 3 and 5. No periprosthetic osteolysis related to polyethylene debris was seen in any radiographs.

Two dislocations occurred postoperatively in 2 (0.9%) patients. Both hips underwent revision surgery to replace the acetabular shell in one case and to replace the acetabular liner in the second case. In both revisions the femoral stem was left in situ. Other complications occurred were 1 (0.5%) intertrochanteric femoral fracture occurred intraoperatively during stem placement and treated successfully with cerclage, 1 groin pain and 1 thigh pain. The number of complications did not differ between stem offset options (Table 3).

**Table 3:** Details of the cases with complications

Case	Age	Gender	FU	Complication	Revision	Stem Offset	Δ GO	GO discrepancy
1	76	M	4,8	Intraoperative intertrochanteric fracture	No	C	0 mm	0 mm
2	69	M	4,2	Dislocation	Yes, acetabular cup	C	+ 10 mm	−5 mm
3	64	M	4,7	Dislocation	Yes, only acetabular liner	B	+ 8 mm	+ 3 mm
4	68	M	4,0	Groin pain	No	C	+ 2 mm	+ 1 mm
5	75	F	3,5	Thigh pain	No	B	+ 10 mm	+ 3 mm

Survival of the whole implant was 98.9% at 6-year follow-up with revision of any component for any reason as the endpoint. Survival of the femoral stem was 100% at 6-year follow-up with revision of the stem for any reason as the endpoint.

## Discussion

The most important finding in this paper is that a double-tapered straight stem with three different offset options can restore the native anatomy and consequently the native biomechanical hip parameters, whatever type of stem is used. Moreover, at a short- to mid-term follow-up the survivorship of the stem is excellent.

Proper restoration of GO and hip center of rotation is crucial to achieve good and long-term results. The restoration of these parameters depends on a correct surgical technique. The hip abductor muscles lever arm is in fact strictly dependent by implant positioning which determines the reconstruction of the center of rotation, as well as GO. Al-Amiry et al. [1] showed that common errors in LL are mainly caused by improper femoral stem positioning, while global FO reduction results from improper positioning of both the femoral stem and the acetabular cup.

According to Bonnin et al. [20] GO should be restored to the native anatomy. Conventional acetabular preparation consists in medialization of the cup on the acetabular floor, leading to medialization of the hip center of rotation (HCR) and decreasing AO. If surgeon compensates the lack of AO by increasing FO, this restores the GO and the abductor lever arm. Conversely, if surgeon only restores FO, without regards to the AO, GO and the abductor lever arm are reduced. Thus, the FO should be increased in order to compensate the lack of AO.

Some authors advocate the potential advantages in restoring the native HCR and therefore preserving AO [21–23]. Potential advantages include the improvement of acetabular bone stock, lower risk of bone or soft tissue impingement and dislocation, and improved hip kinematics. Similarly, disadvantages of preserving the acetabular offset include a potential decrease of the bone coverage of the acetabular component, a higher risk of anterior overhang (a source of iliopsoas tendon impingement), and an increase of the stress applied on the cup and on the femoral head [22, 23]. Therefore, medialization of the cup gives biomechanical advantages according to Pawel’s law and gives lower resulting forces at the head-cup interface [20].

The restoration of GO should be performed in a range within ≤ ± 5 to 10 mm of native values, to gain better clinical scores, an adequate hip range of motion (ROM), gluteus strength and gait kinetics, and lower incidence of LL discrepancy [2, 4]. We agree with the authors, in fact before any surgery preoperative templating is always performed at our department, in order to choose the proper stem for each morphology of femur, limiting a LL discrepancy.

In our series GO and LL were restored within ±5 mm of the native values, leading consequently to good clinical scores. No statistically significant differences in postoperative GO and LL were found in using type B or C stems, therefore the choice of different offset options seems to be related to hip morphology.

Innmann et al. [5] in their comparative study with three stem designs stated that the postoperative LLD was surgeon but not implant specific. Junior surgeons had slightly larger LL discrepancy, compared to senior surgeons (2.4 vs 1.2 mm; *P* = 0.043). However for both senior and junior surgeons, leg length reconstruction was most difficult with the CLS stem with a postoperative LL discrepancy of 2.6 mm (respectively, 1.6 vs 3.6 mm) than with other types of stems, as a short curved stem (Fitmore, Zimmer-Biomet) with a postoperative LLD of 0.8 mm (*P* = 0.002).

Also von Roth et al. [6] in their randomized controlled trial found at 2 weeks a significant difference in postoperative LLD between patients treated with a short curved stem (Fitmore, Zimmer-Biomet) and patients with the CLS stem (respectively, − 0.7 mm and − 2.9 mm, *P* = .01).

The above-mentioned parameters are also related both with hip biomechanics and lower limb gait kinematics. Renkawitz et al. [24] confirmed, with their gait analysis of 60 patients with unilateral THA, that restoration of LL and GO within ±5 mm increase ROM and gait kinematics. Conversely, the authors didn’t found statistically significant distribution with the clinical scores and the restoration of LL and GO. Another gait analysis conducted by Stief et al. [25] showed that restoring GO correlate positively with the Knee Adduction Moment (KAM), so increasing offset, the knee has a greater varus moment during walking. This means that exceeding offset increase can both increase gluteus tension and hip stability, but, on the other hand, could worsen knee function. It is believed that lateral trochanteric pain may be the consequence of increased offset, but Abdulkarim et al. [26] found in their retrospective controlled case series that lateral trochanteric pain is not associated with implant positioning or increased offset.

Intraoperative femoral fractures with the Synthesis stem remain a risk during component implantation. We found 1 intraoperative intertrochanteric fracturing during stem placement. This specific complication related to this type of straight, double tapered, rectangular cross-sectional shaped design of femoral stem which appears to be reduced in our series in comparison with the classic CLS Spotorno design of which literature reported an incidence up to 4% [27, 28] not depending by learning curve, stem offset or stem positioning [29].

This study, although confirming good clinical and radiographic results with GO restoring within 5 mm range, has some limits. First, it’s retrospective with no control group. In some cases the affected hip in the preoperative radiographs were more externally rotated, due to the arthritic condition, thus the femoral neck seems more valgus and consequently the FO is decreased on the AP radiograph. In this study we compared preoperative parameters with postoperative reconstructed parameters. So, our control was the preoperative condition. It would be more interesting to have available a control group with a different design of stem, in order to directly compare the effectiveness in the restoration of GO and LL of different stem design. Finally, only two offset types of the same stem were used. Type A stem was used in only one case of hip dysplasia sequelae, with a short global offset, a mild deformity in neck anteversion and an increased CCD angle. In our series there were no valgus necks that could be matched with stem’s conformation. Probably this offset option doesn’t represent the morphology of some valgus necks, but our series is too restricted to assert it. Further prospective controlled studies with greater series are necessaries to achieve more reliable and reproducible results, but this paper shows encouraging outcomes.

## Conclusions

The restoration of the biomechanical hip parameters after THA leads to satisfactory results. Surgical technique and preoperative planning are the first fundamental steps together with a stem with different offset options. The use of a traditional cementless double-tapered femoral stem, available with 3 offset options with no stem length changes, is effective in restoring the global femoral offset without any clinically significant change in lower limb length. Clinical results and implant survival are encouraging, despite the short follow-up.

## Data Availability

The data that support the findings of this study are available from “Fondazione Lorenzo Spotorno ONLUS” but restrictions apply to the availability of these data, which were used under license for the current study, and so are not publicly available. Data are however available from the authors upon reasonable request and with permission of “Fondazione Lorenzo Spotorno ONLUS”.

## Abbreviations

THA: Total hip arthroplasty
GO: Global offset
FO: Femoral offset
AO: Acetabular offset
LL: Limb length
HHS: Harris hip score
AC: Andrea Camera
CLS: Cementless Lorenzo Spotorno
CCD: Centrum-collum-diaphyseal
AP: Antero-posterior
SB: Stefano Biggi
SD: Standard deviation
ROM: Range of motion
KAM: Knee adduction moment
